# *Castanea sativa* Flower Extract Accelerates Burn Wound Healing via Antioxidant and Anti-Inflammatory Mechanisms in Juvenile Rats

**DOI:** 10.3390/ph19071059

**Published:** 2026-07-09

**Authors:** Şeyma Şimşirgil Kara, Özhan Özcan, Bilge Bal Özkaptan, Özgür Korhan Tunçel, Huriye Demet Cabar, Kıvanç Öncü, Dilek Sağır

**Affiliations:** 1 Faculty of Health Sciences, Sinop University, Sinop 57000, Türkiye; bozkaptan@sinop.edu.tr (B.B.Ö.); dcabar@sinop.edu.tr (H.D.C.); dileks@sinop.edu.tr (D.S.); 2 Department of Intensive Care Unit, Sinop Atatürk Public Hospital, Sinop 57000, Türkiye; ozhanturkey@hotmail.com; 3 Department of Medical Biochemistry, Faculty of Medicine, Ondokuz Mayıs University, Samsun 55270, Türkiye; ozgurkorhan@yahoo.com; 4 Department of Clinical Sciences, Faculty of Dentistry, Ondokuz Mayıs University, Samsun 55270, Türkiye; dr.kivanc.oncu@gmail.com

**Keywords:** anti-inflammatory, burn wound healing, *Castanea sativa* flower, juvenile rat model, oxidative stress, pediatric burn model, polyphenols, silver sulfadiazine

## Abstract

**Background/Objectives:** Burn injuries in children represent a significant clinical challenge, as current standard-of-care agents such as silver sulfadiazine (SSD) present limitations, including delayed re-epithelialization. This study aimed to evaluate the therapeutic potential of *Castanea sativa* (sweet chestnut) flower extract—rich in polyphenols and flavonoids with documented antioxidant, anti-inflammatory, and antimicrobial properties but previously uncharacterized in burn wound healing—applied topically on second-degree burn wounds in a juvenile rat model, comparing its efficacy to SSD and their combination. **Methods:** Forty five-week-old female Wistar albino juvenile rats were randomly allocated into five groups (*n* = 8): burn control (Group C), SSD monotherapy (Group BS), vaseline vehicle/sham (Group Sham), 5% chestnut flower extract (Group BCs), and SSD combined with extract (Group BSCs). All topical treatments were applied once daily for 14 days. Healing outcomes were assessed by macroscopic wound closure analysis, systemic organ stress markers (ALT, AST, BUN), oxidative stress indices (MDA, SOD, CAT, GPx, GSH), inflammatory cytokines (TNF-α, IL-1β, IL-6, IL-10), and histopathological/immunohistochemical analyses (Ki-67, VEGF). **Results:** All active treatment groups demonstrated significant reductions in organ damage markers, oxidative stress burden, and pro-inflammatory cytokine levels, alongside enhanced antioxidant enzyme activity, compared to Group C (*p* < 0.001). Extract-treated groups exhibited more pronounced suppression of oxidative and inflammatory parameters than SSD monotherapy. The combination group (BSCs) achieved optimal wound healing outcomes, including near-complete re-epithelialization, superior collagen organization, and prominent angiogenesis, corroborated by the highest Ki-67 proliferation index and VEGF expression scores (*p* < 0.001). **Conclusions:** *C. sativa* flower extract significantly accelerates burn wound healing via antioxidant and anti-inflammatory mechanisms. When combined with SSD, a synergistic effect is observed that overcomes the re-epithelialization delays associated with SSD monotherapy. These findings support *C. sativa* flower extract as a promising candidate for further preclinical and clinical investigation in pediatric burn management, supporting the ethnopharmacological relevance of this plant in traditional wound care practices; further safety and efficacy validation is required before clinical translation.

## 1. Introduction

Burn injuries in children remain a significant public health challenge due to their high incidence and severe associated complications. Despite advances in modern burn management that have reduced hospitalization rates, pediatric burns continue to be associated with substantial morbidity and mortality, primarily attributable to infection, hypertrophic scarring, and donor site limitations [1]. Beyond the acute clinical course, children who sustain burn injuries frequently face long-term physical and psychosocial sequelae arising from deformities and functional impairments caused by tissue loss. Hypertrophic scar formation not only affects aesthetic outcomes but also restricts mobility and growth potential in the affected area, underscoring the critical importance of optimal wound healing and scar management in pediatric burn treatment [2].

Current trends in wound care research are increasingly focused on the development of multifunctional dressings and bioactive formulations capable of actively responding to the wound microenvironment and exerting targeted anti-inflammatory and antimicrobial effects. For instance, stimuli-responsive hydrogel systems incorporating advanced functional materials have recently been developed to dynamically adapt to wound conditions, offering controlled drug release and enhanced healing outcomes [3]. In parallel, naturally derived bioactive compounds, including plant-based polysaccharides, have attracted growing attention for their anti-inflammatory properties and potential application in wound healing formulations, reflecting a broader shift toward plant-derived therapeutics as safer and more sustainable alternatives to conventional agents [4]. These developments collectively underscore a growing interest in harnessing natural antioxidant and anti-inflammatory compounds, such as those found in *Castanea sativa* flower extract, as promising components of next-generation topical wound care formulations.

Accumulating evidence indicates a strong correlation between wound healing time and scar severity; as the epithelialization period of a burn wound lengthens, scar severity increases, whereas rapid epithelialization significantly reduces hypertrophic scarring [5]. Consequently, the goal of modern burn treatment extends beyond wound closure to encompass the acceleration of the healing process and the minimization of long-term tissue deformities [6].

Silver sulfadiazine (SSD) has long been the standard topical agent in burn wound management [7,8,9]. Although its broad-spectrum antimicrobial efficacy is well established, SSD is associated with several disadvantages, including systemic adverse effects such as leukopenia, crystalluria, and allergic reactions. Furthermore, SSD has been documented to delay re-epithelialization, potentially prolonging wound closure time [8]. These limitations have generated increasing clinical interest in alternative or complementary herbal therapeutic approaches, particularly for pediatric patients [10]. In burn pathophysiology, excessive production of reactive oxygen species (ROS) and the ensuing hyperinflammatory response exacerbate secondary tissue damage and impede healing. Among natural bioactive compounds, *Castanea sativa* Mill. (sweet chestnut; Fagaceae) possesses significant antioxidant, anti-inflammatory, and antimicrobial properties attributable to its rich polyphenolic composition. Phytochemical investigations of *C. sativa* have identified several key bioactive constituents, including phenolic acids such as gallic acid and ellagic acid; flavonoids such as quercetin and rutin; and hydrolyzable tannins, particularly ellagitannins, which are considered major contributors to the plant’s biological activity [11,12]. In line with this, our own phytochemical analysis of the flower extract identified ellagic acid as the predominant phenolic compound, followed by rutin, syringic acid, gallic acid, and quercetin. Extracts derived from chestnut leaves, bark, and flowers have been reported to scavenge ROS, inhibit key inflammatory signaling pathways (NF-κB and MAPK), and exhibit broad-spectrum antimicrobial activity [13]. These properties suggest that chestnut-derived compounds may help modulate inflammation by mitigating oxidative stress in burn wounds while also reducing infection risk [14,15]. This is consistent with emerging evidence that plant-derived bioactive compounds can reverse inflammatory cellular senescence and prevent relapse of inflammatory skin conditions, further supporting the therapeutic relevance of phytochemical-rich extracts for skin and wound-related applications [16]. Additionally, chestnut extracts have been shown to support collagen synthesis by enhancing fibroblast activity and to accelerate re-epithelialization by maintaining moisture balance. However, existing research has been predominantly focused on *C. sativa* wood extract, with no direct investigation of chestnut flower extract effects on burn wound healing reported in the literature to date.

In addition to its experimentally validated biological effects, *C. sativa* has a longstanding tradition of use in ethnomedicine, particularly in rural areas of Turkey’s Black Sea region. In Sinop Province—specifically the Erfelek district—chestnut flowers are traditionally prepared as topical formulations and applied to skin injuries, including minor burns, cuts, and inflammatory skin conditions. Local ethnobotanical knowledge attributes anti-inflammatory, wound-healing, and tissue-regenerating properties to these preparations. However, these specific therapeutic claims have not been validated through controlled pharmacological investigations. There is therefore a compelling rationale to evaluate this plant within a standardized experimental burn model to bridge the gap between traditional knowledge and evidence-based pharmacological validation.

Accordingly, this study aimed to investigate the effects of *Castanea sativa* Mill. (Fagaceae) flower extract—both as monotherapy and in combination with SSD—on burn wound healing in a juvenile rat model, with the goal of elucidating potential synergistic effects. The study evaluated macroscopic wound closure rates, systemic organ function markers, antioxidant parameters, pro- and anti-inflammatory cytokines, and histological and immunohistochemical characteristics of the wound site. These findings are intended to provide experimental evidence supporting a plant-based complementary treatment strategy with translational relevance to pediatric burn management.

## 2. Results

### 2.1. Macroscopic Findings and Wound Closure Analysis

On days 1 and 14 of the experiment, the weights of all animals were measured individually. The weight gain values for the rats in the experimental groups were calculated as mean ± standard deviation (SD). Weight gain was 50.75 ± 4.98 g in the control group (C, *n* = 8); 48.88 ± 2.80 g in the BCs group (*n* = 8); 53.50 ± 3.93 g in the BS group (*n* = 8); 51.38 ± 2.77 g in the BSCs group (*n* = 8); and 51.38 ± 5.04 g in the Sham group (*n* = 8). Weight gain values among the groups were compared using one-way analysis of variance (ANOVA), and no statistically significant difference was found between the groups (F = 1.350; *p* = 0.271; *p* > 0.05).

**Figure 1:**
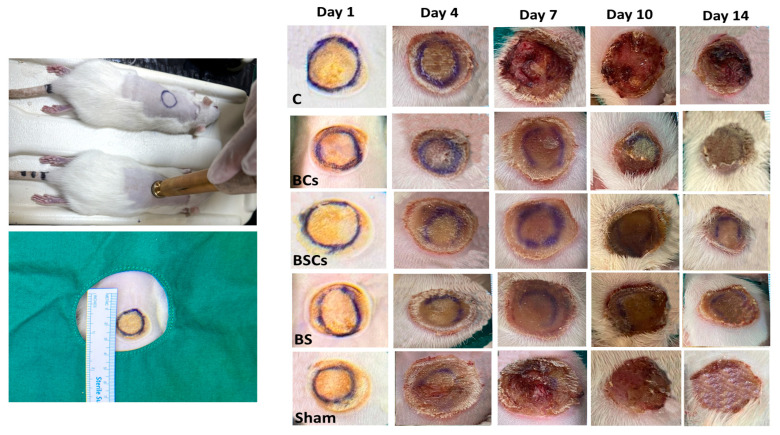
Establishment of the experimental burn model and macroscopic evaluation of the wound healing process. The left panel shows the creation of a standardized second-degree burn wound in rats and the initial appearance (Day 1). The lower left panel presents the standardization and measurement of the wound diameter. The right panel presents representative wound images for the experimental groups (Group C, Group BCs, Group BSCs, Group BS, and Group Sham; see Section 4.7 for group definitions) over time (Days 1, 4, 7, 10, and 14). All images were taken from the same anatomical region and reflect a comparative evaluation of macroscopic changes in the wound area and the healing process.

Macroscopic evaluation of the experimental burn model revealed distinct intergroup differences in wound healing progression (Figure 1). Group BSCs consistently demonstrated faster wound contraction, clearer re-epithelialization, and a structurally more organized tissue appearance compared to all other groups. In contrast, Group C exhibited a persistently wider wound area and sluggish healing progression throughout the study period.

**Figure 2:**
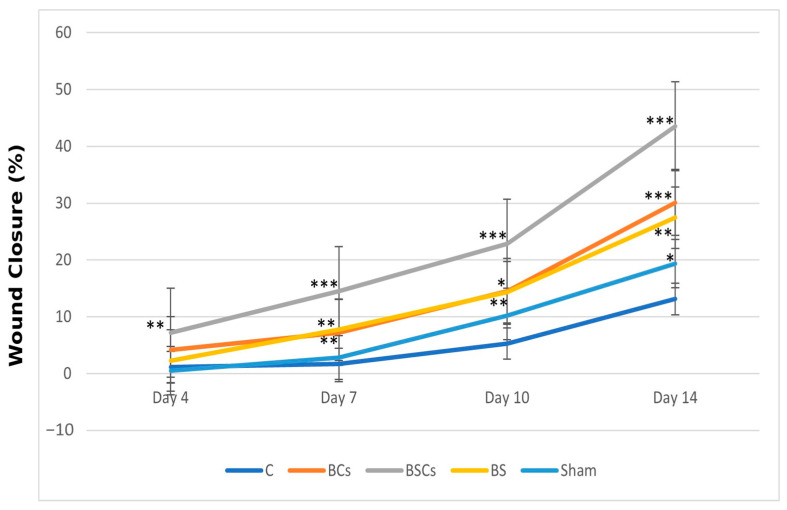
Time-dependent changes in wound closure percentages on days 4, 7, 10, and 14 in the experimental groups. The *Y*-axis represents wound closure (%). All values are presented as mean ± standard deviation (SD). A significant increase in wound closure rates was observed in the treated groups compared to Group C. In particular, Group BSCs demonstrated the highest wound closure rates at all time points. Statistical significance was assessed relative to Group C and is indicated as * *p* < 0.05, ** *p* < 0.01, *** *p* < 0.001.

Quantitative assessment of wound closure percentages on Days 4, 7, 10, and 14 confirmed these macroscopic observations (Figure 2). Although wound healing rates increased over time in all groups, the magnitude of improvement differed significantly between groups. On Day 4, all active treatment groups showed significantly higher wound closure rates compared to Group C, with the most pronounced increase observed in Group BSCs. By Day 7, Group BSCs had surpassed all other groups in wound closure rate, while significant increases persisted in Groups BCs and BS compared to Group C. On Days 10 and 14, Group BSCs consistently maintained the highest closure rate, with Groups BCs and BS also showing significantly higher values than Group C. In Group C, wound closure remained significantly lower throughout the observation period.

### 2.2. Systemic Organ Function Markers

**Figure 3:**
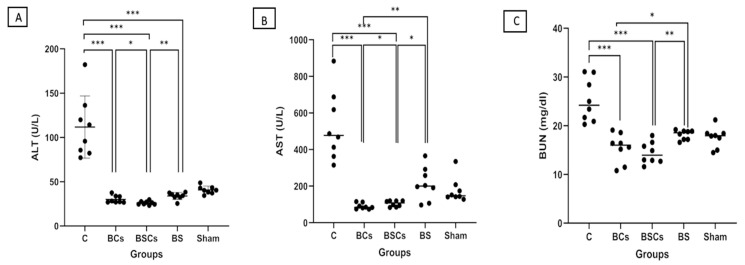
Comparison of systemic liver and kidney function parameters among the experimental groups. Plasma levels of (**A**) ALT, (**B**) AST, and (**C**) BUN are shown. Data are presented as individual data points alongside the mean ± standard deviation (SD). Statistical significance was assessed between the groups and is indicated as * *p* < 0.05, ** *p* < 0.01, *** *p* < 0.001.

Plasma levels of ALT, AST, and BUN—markers of hepatic and renal damage—were highest in Group C. Significant reductions in all three parameters were observed across all active treatment groups compared to Group C (*p* < 0.001 for ALT, AST, and BUN; Figure 3). These reductions were most pronounced in Groups BCs and BSCs. Specifically, Group BSCs showed significantly lower ALT (*p* = 0.003), AST (*p* = 0.016), and BUN (*p* = 0.003) levels compared to Group BS. Additionally, ALT levels were significantly lower in Group BSCs compared to Group BCs (*p* = 0.036), whereas AST levels were significantly lower in Group BCs compared to Group BSCs (*p* = 0.036).

### 2.3. Oxidative Stress and Antioxidant Parameters

**Figure 4:**
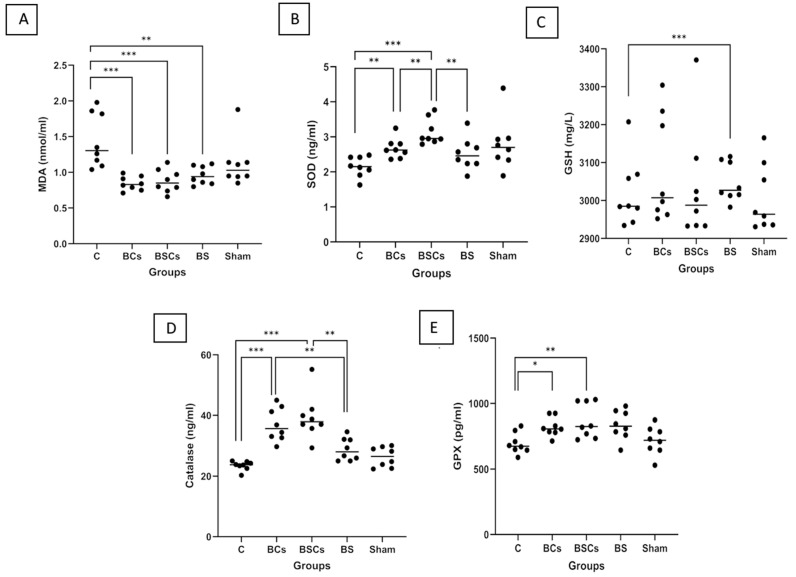
Comparison of oxidative stress and antioxidant parameters in the experimental groups. (**A**) MDA, (**B**) SOD, (**C**) GSH, (**D**) catalase, and (**E**) GPx levels are shown. Data are presented as individual data points along with the mean ± standard deviation (mean ± SD). Statistical significance was assessed between groups and is indicated as * *p* < 0.05, ** *p* < 0.01, *** *p* < 0.001.

MDA levels, a marker of lipid peroxidation, were significantly reduced in all active treatment groups compared to Group C (*p* = 0.001; Figure 4). No significant intergroup differences were detected in GSH levels (*p* > 0.05). Catalase, SOD, and GPx activities were significantly elevated in all treatment groups relative to Group C (*p* < 0.001 for CAT; *p* = 0.001 for SOD; *p* = 0.014 for GPx). The most pronounced increases in antioxidant enzyme activities were observed in Groups BCs and BSCs. Catalase activity was significantly higher in Group BCs than in Group BS (*p* = 0.006). Both catalase (*p* = 0.003) and SOD (*p* = 0.009) activities were significantly higher in Group BSCs compared to Group BS. SOD activity was also significantly higher in Group BSCs compared to Group BCs (*p* = 0.012). No statistically significant differences were detected between Groups BS and BSCs for GPx, MDA, or GSH levels (*p* > 0.05).

### 2.4. Inflammatory Cytokine Levels

**Figure 5:**
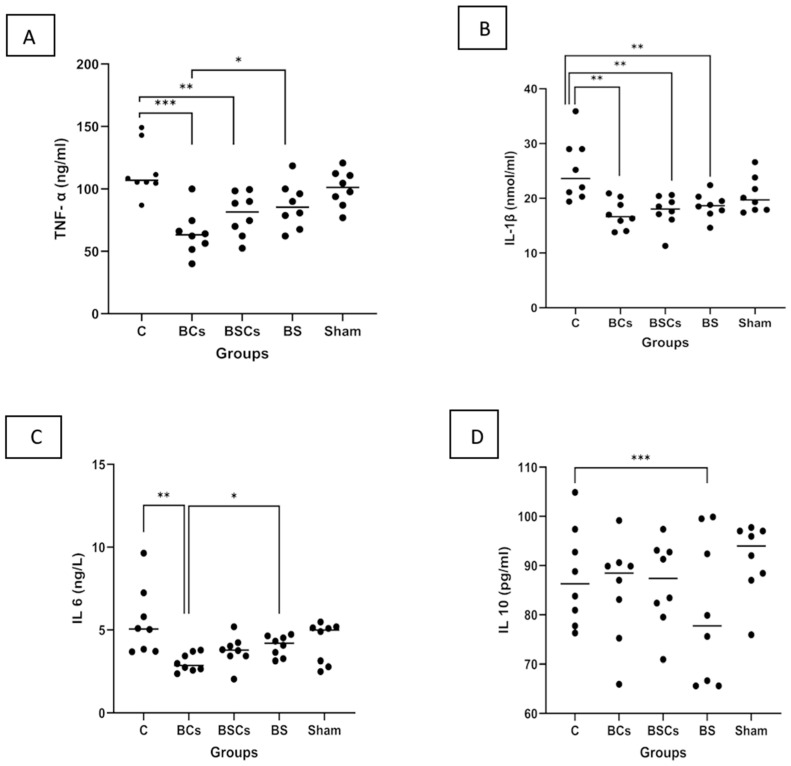
Comparison of systemic inflammatory cytokine levels among the experimental groups. Plasma levels of (**A**) TNF-α, (**B**) IL-1β, (**C**) IL-6, and (**D**) IL-10 are shown. Data are presented as individual data points alongside the mean ± standard deviation (SD). Statistical significance was assessed between the groups and is indicated as * *p* < 0.05, ** *p* < 0.01, *** *p* < 0.001.

Plasma levels of the pro-inflammatory cytokines TNF-α, IL-1β, and IL-6 were significantly elevated in Group C relative to all active treatment groups (*p* < 0.001 for TNF-α; *p* = 0.004 for IL-1β; *p* = 0.013 for IL-6; Figure 5). TNF-α and IL-1β levels in Group C were significantly higher than in both Groups BCs and BSCs. For IL-6, Group C levels were significantly higher than Group BCs. Among active treatment groups, TNF-α (*p* = 0.027) and IL-6 (*p* = 0.012) levels were significantly lower in Group BCs compared to Group BS. Regarding the anti-inflammatory cytokine IL-10, a significant difference was detected only between Group C and Group BS. No significant intergroup differences were observed for TNF-α, IL-1β, IL-6, or IL-10 between Groups BS and BSCs (*p* > 0.05).

### 2.5. Histopathological Findings

**Figure 6:**
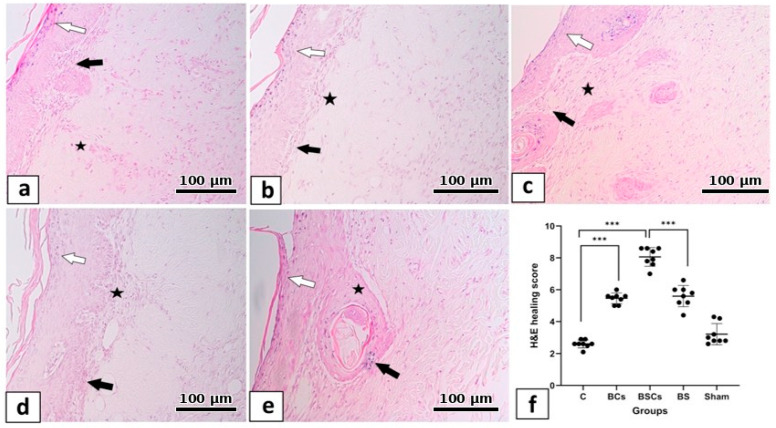
Representative hematoxylin and eosin (H&E)-stained skin sections and semi-quantitative healing score analysis of the experimental groups. Representative images for (**a**) Group C, (**b**) Group BCs, (**c**) Group BSCs, (**d**) Group BS, and (**e**) Group Sham. White arrows indicate re-epithelialization, black arrows indicate granulation tissue formation, and stars indicate inflammatory cell infiltration. (**f**) A comparison of the total H&E histological healing scores among the groups. Data are expressed as mean ± SD. Statistical significance was assessed between the groups and is indicated as (*** *p* < 0.001).

Hematoxylin and eosin (H&E)-stained sections revealed marked intergroup differences in tissue repair parameters (Figure 6). Group C exhibited incomplete re-epithelialization, intense inflammatory cell infiltration, insufficient granulation tissue formation, and limited angiogenesis, resulting in the lowest overall histological healing score. Group BCs showed moderate re-epithelialization and granulation tissue formation with markedly reduced inflammatory cell infiltration relative to Group C, along with moderately increased angiogenesis. Group BS demonstrated more pronounced epithelial regeneration, well-developed granulation tissue, further reduced inflammatory infiltration, and increased neovascularization compared to Group BCs. Group Sham showed mild re-epithelialization and moderate granulation tissue formation; however, inflammatory infiltration remained relatively prominent, and overall healing was lower than in the active treatment groups.

The most pronounced histopathological improvement was observed in Group BSCs, which demonstrated nearly complete epithelialization, dense and well-organized granulation tissue, minimal inflammatory infiltration, and prominent angiogenesis. The total histological healing score for Group BSCs was significantly higher than for all other groups (*p* < 0.001).

**Figure 7:**
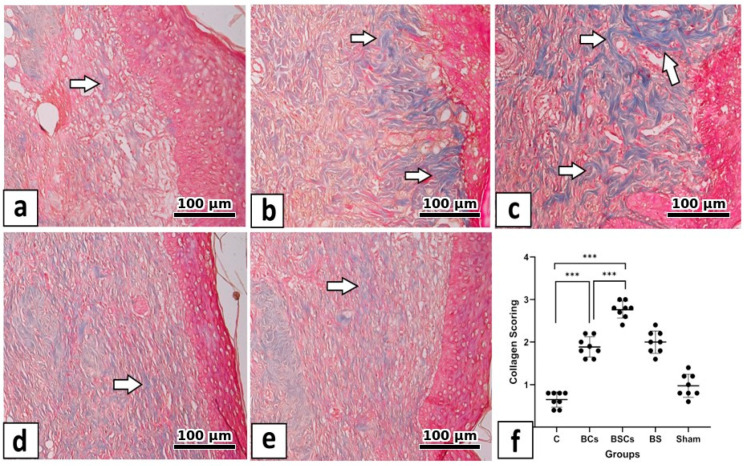
Representative Masson’s trichrome-stained skin sections from the experimental groups and semi-quantitative scoring analysis. Collagen fibers are indicated by white arrows in representative images for (**a**) Group C, (**b**) Group BCs, (**c**) Group BSCs, (**d**) Group BS, and (**e**) Group Sham. (**f**) A comparison of the total Masson’s trichrome histological scores among the groups. Data are expressed as mean ± SD. Statistical significance was assessed between the groups and is indicated as (*** *p* < 0.001).

Masson’s trichrome staining further corroborated these findings (Figure 7). Group C showed minimal and irregularly distributed collagen deposition. Collagen deposition progressively increased in the active treatment groups; Groups BCs and BS exhibited moderately organized collagen fibers, while Group BSCs demonstrated the highest degree of collagen deposition and spatial organization. Group Sham showed mild to moderate collagen deposition, remaining below active treatment group levels.

### 2.6. Immunohistochemical Findings

**Figure 8:**
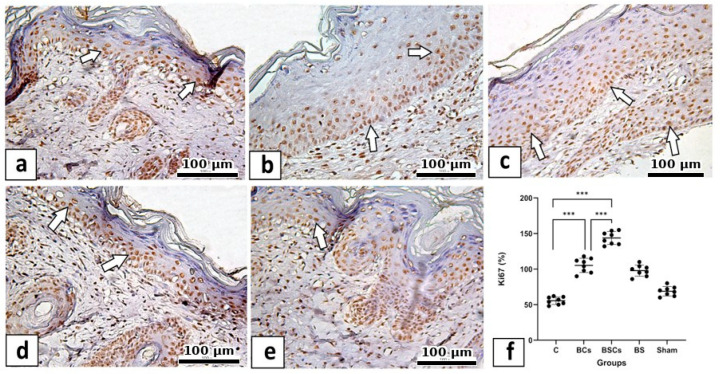
Representative Ki-67 immunohistochemically stained skin sections and proliferation index analysis for the experimental groups. Representative images for (**a**) Group C, (**b**) Group BCs, (**c**) Group BSCs, (**d**) Group BS, and (**e**) Group Sham; Ki-67-positive cells are indicated by white arrows. (**f**) A comparison of proliferation index (Ki-67) scores among the groups. Data are expressed as mean ± SD. Statistical significance was assessed between the groups and is indicated as *** *p* < 0.001.

Ki-67 immunostaining revealed low proliferative activity in Group C (Figure 8). Significant increases in Ki-67-positive cells were observed in all active treatment groups (*p* < 0.001). The highest proliferation index was detected in Group BSCs, followed by Group BS and Group BCs. Group Sham showed a moderate increase compared to Group C but remained significantly lower than active treatment groups (*p* < 0.001).

**Figure 9:**
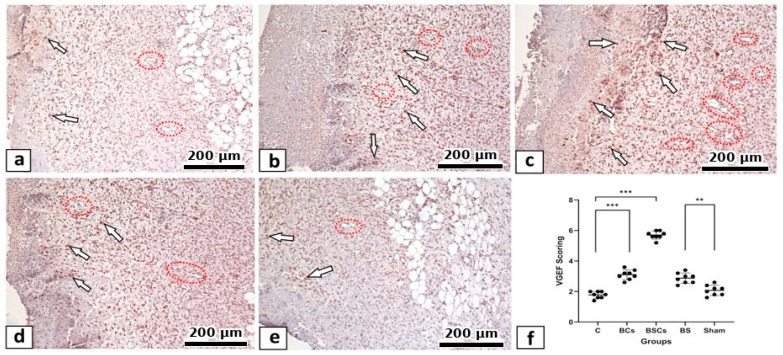
Representative VEGF immunohistochemically stained skin sections and semi-quantitative scoring analysis for the experimental groups. Representative images for (**a**) Group C, (**b**) Group BCs, (**c**) Group BSCs, (**d**) Group BS, and (**e**) Group Sham; VEGF-positive cells are indicated by white arrows, and new blood vessel formation (angiogenesis) by red circles. (**f**) A comparison of VEGF expression scores among the groups. Data are expressed as mean ± SD. Statistical significance was assessed between the groups and is indicated as ** *p* < 0.01 and *** *p* < 0.001.

VEGF immunoreactivity was weak in Group C, indicating limited angiogenic activity (Figure 9). Significant upregulation of VEGF expression was detected in all active treatment groups. The highest VEGF scores were observed in Group BSCs, followed by Group BS and Group BCs. Group Sham showed a slight increase compared to Group C but remained significantly lower than the active treatment groups (*p* < 0.001).

## 3. Discussion

This study comprehensively evaluated the therapeutic effects of chestnut flower extract, SSD, and their combination on burn wound healing, using a multi-parameter approach encompassing systemic organ function, oxidative stress, inflammatory mediators, and histopathological endpoints in a juvenile rat model. The findings demonstrate that burn-induced systemic organ damage, oxidative stress burden, and pro-inflammatory cytokine responses were significantly suppressed in all active intervention groups compared to Group C, with extract-containing groups consistently demonstrating superior outcomes.

Burn injuries trigger a systemic response that extends beyond local tissue damage, adversely affecting hepatic and renal function. It is well documented in the literature that severe burns cause elevation of ALT and AST through hepatocellular damage and increases in BUN through hypovolemia and systemic inflammation [17,18]. The highest levels of these markers observed in Group C are consistent with these established pathophysiological mechanisms. In contrast, the significant reductions in ALT, AST, and BUN across all treatment groups—most pronounced in Groups BCs and BSCs—indicate effective attenuation of burn-related systemic organ stress. These results are consistent with prior reports demonstrating that plant-derived agents with antioxidant and anti-inflammatory properties may limit secondary tissue damage and contribute to the preservation of organ function [19].

Macroscopic wound assessment results aligned with these biochemical improvements, demonstrating significantly accelerated wound closure in all active treatment groups. The temporal increase in wound contraction was most pronounced in Group BSCs, which consistently achieved the highest wound closure rates from Day 4 through Day 14. It is established that macroscopic healing in burn wounds is closely correlated with inflammatory resolution, fibroblast proliferation, collagen synthesis, and re-epithelialization [20]. These data collectively suggest that chestnut flower extract—and particularly its combination with SSD—substantially accelerates the wound healing cascade.

Oxidative stress plays a fundamental pathophysiological role in burn-related tissue injury. Excessive ROS generation suppresses antioxidant defense mechanisms and promotes lipid peroxidation [21]. The low CAT, SOD, and GPx activities alongside elevated MDA levels in Group C confirm severe oxidative stress in the untreated burn condition. Significant increases in catalase, SOD, and GPx activities with concurrent MDA reduction in the active treatment groups strongly support the antioxidant efficacy of chestnut flower extract. Numerous studies have reported that phenolic- and flavonoid-rich plant-based products increase antioxidant enzyme expression and inhibit lipid peroxidation via free radical scavenging mechanisms [22,23]. The phenolic-rich composition of *C. sativa* flowers, documented to confer high antioxidant capacity [12], provides a mechanistic basis for the observed improvements. A prior in vivo study similarly reported that chestnut flower extract attenuated hepatic oxidative stress by reducing lipid peroxidation [24], corroborating our findings in Group BCs.

The superior antioxidant outcomes in Group BSCs—with the greatest increases in CAT, GPx, and SOD and the most profound MDA reduction—suggest an important pharmacodynamic interaction. Although SSD is a potent antimicrobial agent, it has been reported that prolonged topical SSD application can stimulate localized ROS production, potentially delaying the proliferative phase of wound healing [8,25]. The remarkable biochemical improvements in Group BSCs indicate that phenolic-rich chestnut flower extract effectively scavenges these radicals, compensating for the antioxidant limitations of SSD and creating a synergistic microenvironment favorable for tissue repair.

The absence of statistically significant intergroup differences in GSH levels warrants consideration. Glutathione is a rapidly depleted endogenous antioxidant during acute stress conditions that may be rebalanced over time; thus, its levels can vary considerably depending on the measurement timeline and the specific tissue evaluated [26]. The lack of significant GSH variation, contrasted with marked improvements in catalase, SOD, and GPx activities, strongly suggests that the antioxidant mechanism of chestnut flower extract is primarily mediated through upregulation of enzymatic defense systems rather than non-enzymatic pathways.

The severe inflammatory response following burn injury—characterized by excessive release of TNF-α, IL-1β, and IL-6—is intimately linked to oxidative stress and is a key driver of secondary tissue damage [27]. The elevated pro-inflammatory cytokine levels in Group C confirm the severity of burn-induced systemic inflammation in this model. The significant reductions in TNF-α, IL-1β, and IL-6 observed in Groups BCs and BSCs support the potent anti-inflammatory efficacy of the chestnut flower extract. It is well established that plant-derived antioxidants suppress cytokine production through inhibition of key inflammatory signaling pathways, particularly NF-κB [24,28]. The phenolic composition of *C. sativa* flowers likely mediates this immunomodulatory effect through analogous molecular mechanisms.

The absence of significant intergroup differences in IL-10 levels is attributable to the temporal dynamics of the post-burn inflammatory response. The IL-10 response in experimental burn models is highly transient and fluctuates according to measurement timing and tissue specificity [29]. Since measurements were conducted on Day 14—corresponding to the late inflammatory and active proliferative phases—the lack of variation in IL-10 suggests that the anti-inflammatory cytokine response had reached a plateau or returned to baseline. This finding reinforces the notion that the primary immunomodulatory benefit of the treatments at this stage operates through profound suppression of pro-inflammatory mediators rather than continued IL-10 upregulation. This pattern is consistent with broader evidence linking dysregulated pro-inflammatory signaling, including NF-κB-dependent pathways, to impaired tissue repair across various inflammatory conditions [30], while suppression of the IL-23/IL-17/NF-κB inflammatory axis has similarly been shown to attenuate excessive inflammatory responses in other tissue contexts [31], supporting the plausibility of the cytokine suppression mechanism proposed for the extract in the present study.

The histopathological findings provided direct tissue-level evidence of the biochemical improvements. The observation of significant inflammatory infiltration, insufficient granulation tissue formation, and limited angiogenesis in Group C confirms that an uncontrolled inflammatory response impedes tissue repair, consistent with the established literature [32]. In contrast, the active treatment groups demonstrated progressive improvements in all healing parameters, with Group BSCs achieving the most comprehensive repair: near-complete re-epithelialization, dense, well-organized granulation tissue, minimal inflammatory infiltration, and prominent angiogenesis. These outcomes are mechanistically consistent with the combined antioxidant and anti-inflammatory effects of the chestnut flower extract and the antimicrobial properties of SSD acting in a complementary fashion.

Masson’s trichrome findings, revealing the highest collagen deposition and spatial organization in Group BSCs, provide further evidence supporting the extract’s positive effect on fibroblast activity and extracellular matrix synthesis. Robust collagen synthesis and spatial organization are recognized as critical determinants of the proliferative and remodeling phases of wound healing [20,33]. The significant upregulation of Ki-67 expression—highest in Group BSCs—substantiates accelerated re-epithelialization and cellular proliferation, as Ki-67 is an established nuclear marker of active cell cycle phases and a reliable proxy for epithelial and fibroblast proliferation during wound repair [34]. Correspondingly, the peak VEGF expression in Group BSCs indicates robust angiogenic activation. VEGF is a principal regulator of neovascularization, supporting tissue regeneration by facilitating oxygen and nutrient delivery to the wound bed [35,36].

Although SSD is widely employed in clinical burn management due to its potent antibacterial efficacy, reports of potential delays in re-epithelialization and inhibition of cellular proliferation represent significant limitations [8]. SSD exerts its antibacterial activity primarily through silver ion-mediated disruption of bacterial cell membranes and inhibition of microbial DNA replication; however, this mechanism is non-selective and has also been associated with cytotoxic effects on host keratinocytes and fibroblasts, contributing to the delayed re-epithelialization observed with SSD monotherapy [8]. We propose that the synergistic effect observed in Group BSCs arises from a complementary, rather than overlapping, mechanism of action: while SSD provides direct antimicrobial control of the wound bed, the phenolic and flavonoid constituents of the extract—notably ellagic acid and rutin, identified in Section 4.4—may concurrently mitigate SSD-associated cytotoxicity by scavenging reactive oxygen species and suppressing NF-κB-mediated inflammatory signaling in proliferating keratinocytes and fibroblasts. This dual action—antimicrobial control via SSD combined with antioxidant/anti-inflammatory protection of proliferating cells via the extract—may explain why Group BSCs achieved superior re-epithelialization, collagen organization, and angiogenesis compared to SSD monotherapy (Group BS), effectively overcoming the proliferative limitations typically associated with SSD. The markedly superior outcomes in Group BSCs compared to Group BS suggest that chestnut flower extract effectively counteracts these limitations, compensating for the potential cytotoxic effects of SSD on proliferating cells and thereby maximizing the therapeutic benefit of the combined approach. Recent literature has demonstrated that chestnut flower extract promotes dermal fibroblast migration under inflammatory conditions, offering significant wound healing benefits through its anti-inflammatory, antioxidant, and antibacterial properties [15]. These data, together with the well-documented roles of natural medicinal plants in the treatment of skin damage [37,38,39] and their widespread traditional application for skin injuries [40,41], collectively validate the translational potential of this combination approach.

From a translational standpoint, the development of *C. sativa* flower extract into a clinically applicable pharmaceutical product will require further formulation and standardization work beyond the vaseline-based ointment evaluated in this study. Several delivery system strategies may be considered to enhance the extract’s stability, skin penetration, and controlled release at the wound site. Hydrogel-based formulations, for instance, could provide a moist wound-healing environment while facilitating sustained release of the phenolic constituents [3]. Nanoparticle-based delivery systems, such as polymeric nanoparticles or liposomal encapsulation, may improve the bioavailability and stability of labile phenolic compounds such as ellagic acid and rutin, which are susceptible to oxidative degradation upon prolonged topical exposure [42]. Similarly, incorporation of the extract into nanoemulsions or microsponge-based gels could enhance dermal penetration while minimizing systemic absorption, an important consideration given the thin and permeable skin barrier characteristic of pediatric patients [43].

Standardization of the active components will also be essential for the reproducible pharmaceutical development of this extract. Quantification of the active phenolic constituents, as performed in the present study via RP-HPLC-PDA (Section 4.4), provides a foundation for establishing batch-to-batch quality control criteria, with ellagic acid representing a candidate marker compound given its predominance in the extract (2065.49 ± 8.69 µg/g) and its well-documented antioxidant and anti-inflammatory bioactivity. Future pharmaceutical development would benefit from establishing minimum acceptable thresholds for total phenolic content and key marker compounds, in accordance with pharmacopeial standards for herbal extract quality control, as well as from stability testing under varying storage conditions to determine appropriate shelf-life parameters. It should be noted that the present study evaluated a single extract concentration (5% *w*/*w*), which represents a limitation in determining the optimal therapeutic dose; a systematic dose–response evaluation was beyond the scope of the current study but is planned for future work. Pharmacokinetic and dermal permeation studies, together with such dose-ranging investigations, would further support the rational design of an optimized topical formulation for clinical translation. Such pharmacokinetic evaluations could draw on methodological approaches recently applied to characterize the systemic disposition and interaction profiles of other herbal-derived bioactive compounds [44]. An additional limitation of the present study is the single early endpoint (Day 14), which captures the acute and subacute phases of wound healing but does not allow assessment of long-term outcomes such as scar quality, hypertrophic scarring, or final cosmetic and functional results. Given the clinical importance of scar outcomes in pediatric burn management, future studies incorporating extended follow-up periods and validated scar assessment tools (e.g., the Vancouver Scar Scale) are warranted to comprehensively evaluate the long-term impact of the extract on scar formation and quality.

## 4. Materials and Methods

### 4.1. Animals and Ethical Statement

The study protocol was reviewed and approved by the Ondokuz Mayıs University Institutional Animal Care and Use Committee (IACUC) (Ethics Code: 2025/24). All experimental procedures were conducted in accordance with institutional and international ethical standards for the care and use of laboratory animals, the ARRIVE (Animal Research: Reporting of In Vivo Experiments) guidelines, the European Union Directive 2010/63/EU, and the NIH Guide for the Care and Use of Laboratory Animals. Sample size was determined using G*Power 3.1 software. Based on expected effect size, a significance level of 0.05 (α), and statistical power of 0.80 (1 − β), a minimum of 6 animals per group was required; this was increased to 8 per group to account for potential experimental losses and to enhance statistical robustness. A total of 40 five-week-old female Wistar albino juvenile rats (100–130 g) were obtained from the Ondokuz Mayıs University Research and Application Center for Laboratory Animals. The five-week-old age corresponds to the weaning stage in rats, which is considered analogous to the childhood period in humans [45]. Animals were housed under controlled conditions (22 ± 2 °C, 55–60% relative humidity, 12 h light/dark cycle) with ad libitum access to standard laboratory chow and tap water.

### 4.2. Preparation of Castanea sativa Flower Extract

*Castanea sativa* Mill. flowers were collected in June from the Erfelek district of Sinop Province during peak flowering season. Taxonomic identification was performed by field experts from the Department of Biology, Sinop University. Flowers were dried under shade in a well-ventilated environment for 5–7 days and ground to a fine powder (particle size 500–1000 µm). A 70% ethanol solution (ethanol:water, 70:30 *v*/*v*) was used as the extraction solvent at a plant material-to-solvent ratio of 1:10 (*w*/*v*), selected to maximize phenolic and flavonoid yield [46]. Extraction was performed by stirrer-assisted maceration at room temperature for 24–48 h using a magnetic stirrer, followed by filtration through Whatman filter paper. The solvent was removed under reduced pressure at 40–50 °C using a rotary evaporator, yielding a solvent-free dry extract suitable for biomedical applications [46]. The extract was applied in a vaseline-based ointment formulation at 5% (*w*/*w*) concentration.

### 4.3. Determination of Total Phenolic and Flavonoid Content

The total phenolic content of the extract was determined using the Folin–Ciocalteu method [47], which provides information on the total amount of phenolic-character compounds present in the sample, including phenolic acids, anthocyanins, flavonoids, and tannins. The results were expressed as mg gallic acid equivalents (GAE) per mL of extract. Total flavonoid content was determined according to the procedure described by Fukumoto and Mazza (2000) [48] using quercetin as the reference standard; results were expressed as mg quercetin equivalents (QE) per gram of sample. The total polyphenol and flavonoid content of the *Castanea sativa* flower extract is presented in Table 1.

### 4.4. Determination of Phenolic Compounds by RP-HPLC-PDA

Prior to phenolic content analysis, the ethanolic extract was dried using a rotary evaporator (IKA-Werke, Staufen, Germany), and the residue was redissolved in distilled water adjusted to pH 2 with HCl. Liquid–liquid extraction was performed sequentially three times using 5 mL of diethyl ether and ethyl acetate, respectively. The organic phase was collected, and the solvent was evaporated. The resulting residue was redissolved and injected into the HPLC system (Shimadzu Corporation LC-20AT, Kyoto, Japan).

Phenolic content analysis of the samples was performed using an RP-HPLC-PDA system. The mobile phase consisted of acetonitrile: water (70:30) and water containing 2% acetic acid. The injection volume was 20 µL, the flow rate was 1 mL/min, and the column temperature was set to 30 °C. All analyses were performed in three independent replicates (*n* = 3), and results were expressed as mean ± standard deviation in µg of standard phenolic compound per gram of sample. The phenolic compound content of the extract is presented in Table 2.

Ellagic acid was identified as the predominant phenolic compound in the extract, followed by rutin, syringic acid, gallic acid, and quercetin.

### 4.5. Antimicrobial Activity

The antimicrobial efficacy of the flower extract was screened against *Staphylococcus aureus*, *Bacillus cereus*, *Escherichia coli*, and *Candida albicans*. Antimicrobial activity was evaluated using the agar well diffusion method. Wells of 6 mm diameter were punched in the agar medium, and 50 µL of extract was added to each well. Plates were incubated for 18 h for bacterial strains and 36 h for *Candida* species.

Minimum inhibitory concentration (MIC) values were determined using Brain Heart Infusion Broth (BHIB) for *M. smegmatis* and Mueller–Hinton Broth-II for the other bacterial strains [49]. MIC determination for *Candida* species was performed using RPMI 1640 medium containing 0.2% glucose [50]. Minimum bactericidal concentration (MBC) was determined by sub-culturing 50 µL from the MIC well and the three preceding wells onto plates containing the appropriate medium, followed by incubation at 37 °C for the species-specific incubation period [49]. The results of the antimicrobial screening are presented in Table 3.

The test was repeated three times, and the values represent the mean of the zone diameters. Control groups were Gentamicin (0.2 mg/mL) for Gram-negative bacteria, Ampicillin (0.2 mg/mL) for Gram-positive bacteria, and Amphotericin B (0.2 mg/mL) for fungal species. MIC: Minimum Inhibitory Concentration; MBC: Minimum Bactericidal Concentration.

### 4.6. Preparation of Ointments and Standard Treatments

To prepare the 5% (*w*/*w*) active ointment, pharmaceutical-grade white soft paraffin (vaseline) was heated in a water bath to 40–45 °C. Then, 5 g of dry chestnut flower extract was incorporated into 95 g of softened vaseline in small increments, homogenized using a magnetic stirrer, and—upon cooling—transferred to sterile glass jars. Sterile spatulas were used for each application to prevent contamination. The sham group received pure pharmaceutical-grade vaseline without active extract. The positive control group received commercially available 1% SSD cream (Silverdin^®^, Deva Holding A.Ş., Istanbul, Türkiye) in accordance with manufacturer instructions.

### 4.7. Burn Model and Experimental Groups

General anesthesia was induced by intraperitoneal administration of ketamine (50 mg/kg; Ketalar^®^, Eczacıbaşı, Istanbul, Türkiye) and xylazine (10 mg/kg; Rompun^®^, Bayer, Istanbul, Türkiye). A standardized second-degree burn model was established following the method described by Kulaç et al. [51]: a circular brass disk (5 mm thick) was heated to 100 ± 1 °C on a thermostat-controlled hot plate, temperature-verified with an infrared thermometer, and applied to the shaved dorsal skin for 30 s under its own weight. The affected area was rinsed with saline, and paracetamol (approximately 2 mg/mL; Paracerol^®^, Polifarma, Istanbul, Türkiye) was added to drinking water for the first 24 h post-procedure for analgesia.

Forty rats were randomly allocated by an independent researcher into five groups (*n* = 8): (C) burn control—no topical treatment; (BS) burn + 1% SSD cream applied once daily; (Sham) burn + vaseline applied once daily; (BCs) burn + 5% chestnut flower extract ointment applied once daily; and (BSCs) burn + 1% SSD cream (morning) and 5% chestnut flower extract ointment (evening, ~12 h apart). All wound sites were covered with sterile gauze pads. A rigorous double-blind protocol was implemented: treatment administrators were aware of group assignments, while investigators performing biochemical, histopathological, and immunohistochemical analyses were fully blinded. All samples were coded, and blinding was maintained until statistical analysis was complete.

### 4.8. Macroscopic Wound Assessment

Wound healing was assessed macroscopically on Days 1, 4, 7, 10, and 14 by standardized digital photography (fixed distance, fixed lighting, with a millimeter ruler for calibration). Wound areas (mm^2^) were measured using ImageJ software version 1.53 (National Institutes of Health, Bethesda, MD, USA) [52]. Wound closure percentage was calculated as: Wound closure (%) = [(A_0_ − A_t_)/A_0_] × 100, where A_0_ is the initial wound area (Day 1) and A_t_ is the wound area at the given time point. All measurements were performed under blinded conditions, and results were expressed as mean ± SD.

### 4.9. Sample Collection and Biochemical Analyses

On Day 14, all animals were deeply anesthetized (ketamine 90 mg/kg + xylazine 10 mg/kg, intraperitoneal) and sacrificed by intracardiac perfusion following cardiac blood collection (3–4 mL). Blood samples were collected in EDTA tubes and centrifuged at 2000 rpm for 15 min at +4 °C (Hettich Zentrifugen, Tuttlingen, Germany); plasma was separated, aliquoted, and stored at −80 °C. Wound site tissues were excised with healthy tissue margins; half were fixed in 10% neutral-buffered formalin for histological analysis, and the remainder were washed with cold PBS, placed in sterile cryotubes, and stored at −80 °C for biochemical analyses. Plasma ALT, AST, and BUN levels were measured using an automated clinical chemistry analyzer. Plasma cytokines (IL-1β, IL-6, IL-10, TNF-α) were quantified using commercial ELISA kits (BT Lab, Shanghai, China) following manufacturer instructions. Oxidative stress markers—MDA (lipid peroxidation index), SOD, CAT, GPx (antioxidant enzyme activities), and GSH (redox balance marker)—were assessed spectrophotometrically (GENESYS 10S, Thermo Scientific, Waltham, MA, USA) from plasma samples [53,54,55,56].

### 4.10. Histopathological Analysis

Tissue samples were fixed in 10% neutral-buffered formalin for 48 h, processed through graded alcohols and xylene, and embedded in paraffin. Serial 5 µm sections were mounted on slides and stained with hematoxylin and eosin (H&E) for general histological evaluation and with Masson’s trichrome for collagen assessment. Wound healing parameters (re-epithelialization, granulation tissue formation, inflammatory cell infiltration, angiogenesis) were semi-quantitatively scored on a 0–3 scale (0: absent, 1: mild, 2: moderate, 3: marked) by two independent blinded observers using a light microscope (Leica DM2500, Leica Microsystems, Wetzlar, Germany). Collagen deposition was scored 0–3 (0: absent/minimal, 1: thin/loosely arranged, 2: moderate with partial organization, 3: dense/well-organized).

### 4.11. Immunohistochemical Analysis

Immunohistochemical staining was performed using the streptavidin–biotin–peroxidase method [57]. Paraffin-embedded sections (5 µm) were deparaffinized, rehydrated, and subjected to antigen retrieval (citrate buffer pH 6.0, microwave). Endogenous peroxidase was blocked with 3% hydrogen peroxide (10 min), followed by protein blocking. Sections were incubated overnight at 4 °C with primary antibodies: Ki-67 (rabbit monoclonal; Abcam, Cambridge, UK; ab16667) and VEGF (rabbit polyclonal; Abcam; ab46154). After washing, sections were incubated with biotinylated secondary antibody and streptavidin–HRP complex (UltraVision Detection System, Thermo Fisher Scientific, Waltham, MA, USA). Immunoreactivity was visualized using 3,3′-diaminobenzidine (DAB; Dako, Glostrup, Denmark), and nuclei were counterstained with hematoxylin. Negative controls were processed without the primary antibody. Ki-67 immunoreactivity was expressed as a proliferation index (percentage of positively stained nuclei per ≥ 500 counted cells across ≥ 5 high-magnification fields). VEGF expression was semi-quantitatively scored by summing staining intensity (0–3) and proportion of positive cells (0–3), yielding a total score of 0–6. All evaluations were performed by two independent, blinded observers.

### 4.12. Statistical Analysis

Statistical analyses were performed using IBM SPSS (version 26.0, IBM Corp., Armonk, NY, USA) Statistics (IBM Corp., Armonk, NY, USA). Continuous variables are expressed as mean ± standard deviation. Data normality was assessed with the Shapiro–Wilk test. For normally distributed parameters, one-way ANOVA was applied, followed by the Tukey post hoc test for pairwise comparisons. For non-normally distributed data, the Kruskal–Wallis test was used, followed by the Mann–Whitney U test for pairwise comparisons. Statistical significance was set at *p* < 0.05.

## 5. Conclusions

The present study demonstrates that topical application of *Castanea sativa* flower extract significantly accelerates burn wound healing at both the biochemical and histopathological levels by effectively modulating the severe inflammatory response and oxidative stress burden in a juvenile rat model. The marked improvements in systemic organ damage markers and a more favorable biochemical profile compared to SSD monotherapy underscore the extract’s potent anti-inflammatory and systemic protective properties. Most notably, the combination of chestnut flower extract with SSD produced a synergistic therapeutic effect—yielding the most comprehensive tissue regeneration, robust collagen organization, and pronounced angiogenesis—clearly indicating complementarity between their distinct mechanisms of action. SSD’s potent antimicrobial activity, combined with the extract’s antioxidant and anti-inflammatory properties, effectively overcomes the re-epithelialization delays associated with SSD monotherapy. These compelling preclinical findings support *C. sativa* flower extract as a promising candidate for further investigation in burn wound management, simultaneously confirming the ethnopharmacological significance of this plant in traditional wound care practices. As these conclusions are based on a single preclinical animal study, further validation of safety and efficacy—including dose–response evaluation, long-term scar outcome assessment, and ultimately well-controlled clinical trials—is required before any clinical application can be considered. Future investigations incorporating phytochemical characterization of the extract, dose–response evaluation, and serial temporal assessment of molecular pathways will further elucidate the full clinical potential of this synergistic approach.

## Figures and Tables

**Table 1:** Total polyphenol and flavonoid content of *Castanea sativa* flower extract.

Sample	Total Polyphenol (mg GAE/g)	Total Flavonoid (mg QE/g)
*C. sativa* flower extract	52.13 ± 2.40	6.67 ± 0.53

Values are expressed as mean ± standard deviation.

**Table 2:** Phenolic compound content of *Castanea sativa* flower extract determined by RP-HPLC-PDA.

Phenolic Compound	Amount (µg/g)
Gallic acid	287.91 ± 4.44
Syringic acid	352.99 ± 9.11
Rutin	546.57 ± 5.32
Quercetin	150.56 ± 1.93
Ellagic acid	2065.49 ± 8.69

Values are expressed as mean ± standard deviation (µg/g).

**Table 3:** Antimicrobial activity of *Castanea sativa* flower extract against tested microorganisms.

Microorganism	Zone (mm)	MIC (µg/mL)	MBC (µg/mL)	Control (mm)
*S. aureus*	16.69 ± 0.17	30.50	65.50	29.33 ± 1.15
*B. cereus*	10.50 ± 0.50	520	1100	29.00 ± 1.00
*C. albicans*	3.20 ± 0.20	115	280	2.0 ± 0.30
*E. coli*	18.33 ± 0.31	30.00	1260.00	30.00 ± 1.00

Values are expressed as mean ± standard deviation. MIC: minimum inhibitory concentration; MBC: minimum bactericidal/fungicidal concentration.

## Data Availability

The data presented in this study are available on request from the corresponding author due to ethical and institutional restrictions.
